# Acute Feasibility of Vacuum-Assisted Catheter-Based Left Atrial Appendage Inversion in a Swine Model

**DOI:** 10.3390/bioengineering13070777

**Published:** 2026-07-03

**Authors:** Muhammad Ali, Brad Farrell, Khaldoun Ali

**Affiliations:** 1 Kardiologische Praxis, Kattowitzer Str. 191 B, 38226 Salzgitter, Germany; 2 Veranex Preclinical Services, 380 Michel St B, Atlanta, GA 30313, USA; brad.farrell@veranex.com; 3 Department of Cardiothoracic and Vascular Surgery, Städisches Klinikum Braunschweig, Fichtengrund 1, 38126 Braunschweig, Germany; chaldun@hotmail.de

**Keywords:** atrial fibrillation, catheter-based intervention, left atrial appendage, stroke prevention, vacuum-assisted inversion

## Abstract

Background: The left atrial appendage (LAA) is the predominant site of thrombus formation in atrial fibrillation. Current percutaneous LAA occlusion devices require permanent implants. A catheter-based, non-implant mechanical inversion strategy may offer an alternative approach to stroke prevention. Objectives: To assess the feasibility of vacuum-assisted catheter-based inversion of the LAA using transseptal aspiration in a swine model. Methods: A 59-kg domestic swine underwent transseptal access via the right femoral vein under fluoroscopy, transesophageal echocardiography (TEE), and intracardiac echocardiography (ICE). A 22-F aspiration catheter was advanced into the left atrium and positioned at the LAA apex. Negative pressure was generated manually with a 60-mL syringe attached to the aspiration port, and sequential suction–traction maneuvers were performed to induce LAA inversion. Procedural feasibility, hemodynamic stability, imaging changes, and gross pathology were assessed. Results: LAA suction and inversion were feasible. Sequential negative pressure applications resulted in complete inversion, confirmed by multiplane TEE. A mild, non-hemodynamically significant pericardial effusion occurred. Necropsy showed focal apex injury consistent with catheter stiffness and suction forces. Conclusions: Catheter-based vacuum-assisted LAA inversion was technically feasible in this acute swine experiment. However, chronic survival studies are required to evaluate durability of inversion, tissue healing, thrombogenicity, and long-term safety before clinical translation can be considered.

## 1. Introduction

The left atrial appendage (LAA) is a small, muscular outpouching of the left atrium and the predominant site of thrombus formation in patients with non-valvular atrial fibrillation [1,2]. Loss of coordinated atrial contraction leads to blood stasis within the LAA and predisposes to thrombus, accounting for more than 90% of thrombi in this setting [3]. For most patients, the benefit from anticoagulation outweighs the associated increase in the risk of bleeding. However, major challenges to long-term therapy include a substantial ongoing hazard of major bleeding, noncompliance, side effects [4,5,6,7,8,9], and—particularly during the early adoption of novel oral anticoagulants—the absence or restricted availability of specific reversal agents. Percutaneous or surgical LAA occlusion provides an alternative to long-term oral anticoagulation but depends on permanent implants and may be complicated by device-related thrombus, migration, incomplete sealing, or pericardial effusion [10,11,12].

Mechanical inversion of the LAA has recently been introduced as a novel non-implant strategy for stroke prevention. In a proof-of-concept study in swine, Wang and colleagues demonstrated that direct mechanical inversion of the LAA results in anatomic exclusion of the appendage and predictable healing and fibrosis during chronic follow-up [13]. However, their technique required direct manipulation of the appendage wall through surgical or minimally invasive access and did not test any fully catheter-based approach.

We hypothesized that negative pressure delivered through a transseptal aspiration catheter could generate inward traction sufficient to invert the LAA apex without surgical manipulation or implants. In this pilot study, we sought to evaluate the feasibility of vacuum-assisted, catheter-based LAA inversion in a large-animal model and to describe the procedural characteristics and immediate safety profile of this approach.

## 2. Methods

### 2.1. Animal Preparation

A 59-kg domestic Yorkshire swine underwent general anesthesia, endotracheal intubation, and mechanical ventilation according to institutional laboratory animal protocols for acute procedures. Continuous electrocardiographic and hemodynamic monitoring was maintained throughout the experiment. All procedures were approved by the Institutional Animal Care and Use Committee (IACUC). The study was conducted in accordance with ARRIVE guidelines and institutional animal care standards.

### 2.2. Baseline Imaging

**Figure 1:**
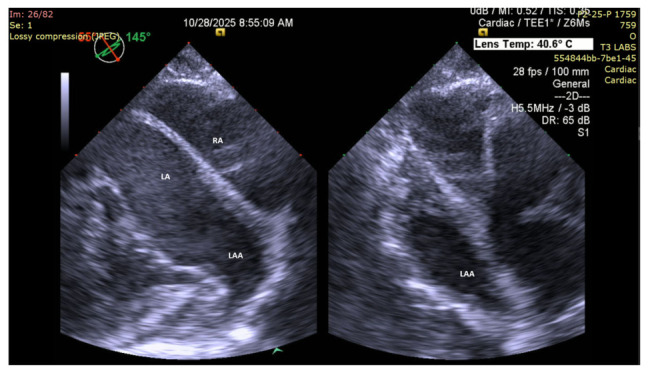
Baseline TEE views of the left atrial appendage. Mid-esophageal two-dimensional views at approximately 55° and 145° show smooth LAA contours without thrombus, confirming suitability for interventional manipulation.Green and red markers indicate imaging orientation and probe angle during TEE acquisition.

Prior to catheterization, a comprehensive TEE examination (Philips Healthcare, Andover, MA, USA) was performed to evaluate global cardiac function, confirm normal atrial anatomy, and exclude baseline LAA thrombus or other anomalies [1]. Standard mid-esophageal views were obtained at multiple angles, including approximately 55° and 145°, to visualize the LAA. Representative baseline TEE images are shown in Figure 1.

### 2.3. Vascular Access and Transseptal Puncture

Right femoral venous access was obtained via surgical cutdown, and a large-bore vascular introducer sheath was inserted. A steerable transseptal sheath (Agilis, Abbott, Abbott Park, IL, USA) was advanced into the right atrium. Under combined ICE (ViewFlex Xtra, Abbott, Abbott Park, IL, USA) and fluoroscopic guidance (Philips Healthcare, Best, The Netherlands), a transseptal puncture was performed at the fossa ovalis using standard techniques. The dilator and sheath were advanced into the left atrium, and an extra-stiff 0.035-inch guidewire (Lunderquist, Cook Medical, Bloomington, IN, USA) was positioned in the left atrium to facilitate subsequent device delivery.

### 2.4. Aspiration Catheter Delivery and Suction Protocol

**Figure 2:**
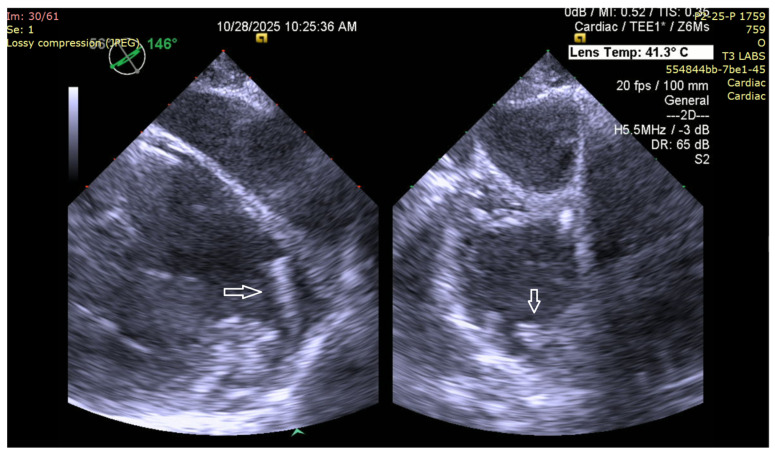
TEE-guided positioning of the aspiration catheter within the LAA. Mid-esophageal views at approximately 56° and 146° demonstrate the catheter tip in the appendage cavity (arrows). At the onset of suction, partial inward deformation of the LAA wall is observed. Green markers indicate imaging orientation and probe angle during TEE acquisition.

**Figure 3:**
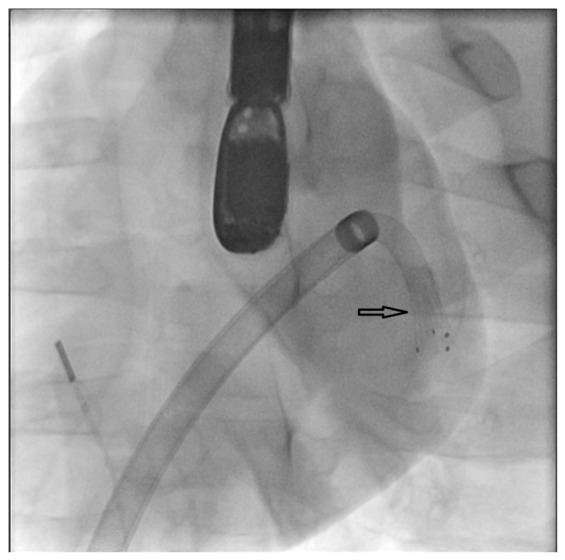
Fluoroscopic guidance of catheter positioning. The distal curve of the aspiration catheter is directed toward the LAA cavity (arrow), confirming appropriate orientation for vacuum-assisted inversion.

The interatrial septal tract was predilated with the dilator from a 22-F AngioVac aspiration catheter (F1885, AngioDynamics, Latham, NY, USA). The AngioVac aspiration guide catheter was then advanced across the septum over the extra-stiff guidewire and positioned within the left atrium under continuous fluoroscopic, ICE, and TEE guidance. The distal curve of the catheter was oriented toward the LAA cavity, and the tip was advanced to the LAA apex (Figure 2 and Figure 3).

Negative pressure for vacuum-assisted inversion was generated manually with a 60-mL catheter-tip syringe connected to the catheter’s aspiration port. After the onset of suction, gentle traction was applied to the catheter while maintaining its orientation to draw the LAA apex inward toward the left atrium. Suction–traction maneuvers were repeated as needed, up to four attempts, until sustained inversion was observed. Suction was manually generated using a 60 mL syringe pulled to maximal volume capacity to create strong negative pressure. The aspiration maneuver was repeated approximately four times, with each suction application lasting approximately 20–30 s. Traction was applied carefully and gently under continuous imaging guidance to minimize tissue injury. Quantitative measurement of traction force was not available in this preliminary feasibility experiment.

### 2.5. Outcome Measures and Necropsy

**Figure 4:**
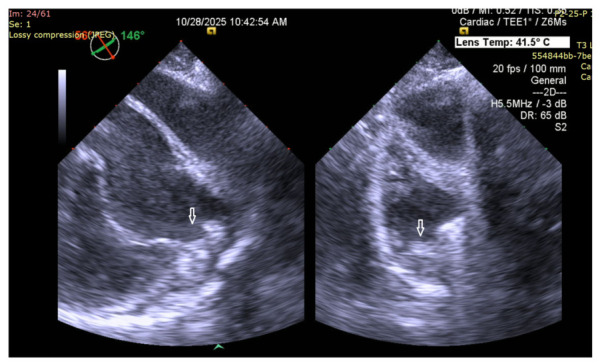
Echocardiographic confirmation of complete LAA inversion. With continued suction, TEE views at approximately 56° and 146° show complete inversion of the LAA into the left atrium (arrows), forming a smooth tissue mound consistent with mechanical inversion from negative pressure.

The primary endpoint was technical success, defined as complete inversion of the LAA apex into the left atrium confirmed by TEE in orthogonal views (Figure 4). Secondary observations included procedural feasibility, hemodynamic stability, occurrence of pericardial effusion, and visible complications on imaging. Following completion of the procedure, the animal was euthanized, and a detailed necropsy was performed. The heart was inspected for evidence of LAA inversion, tissue injury, perforation, or pericardial hemorrhage.

## 3. Results

Baseline TEE confirmed normal cardiac anatomy and a smooth-contoured LAA without evidence of thrombus. Transseptal puncture and advancement of the 22-F aspiration catheter into the left atrium were successfully performed over the extra-stiff guidewire.

During the initial suction attempts, inward deformation of the LAA wall was observed on TEE and ICE, but the appendage returned to its native configuration once suction was released. On the fourth attempt, sustained inward displacement of the appendage apex was achieved. Multiplane TEE imaging demonstrated a smooth, homogeneous tissue mound protruding into the left atrial cavity, consistent with complete inversion of the LAA.

Contrast injection through the aspiration catheter during the later stages of the procedure revealed a mild pericardial effusion, which was confirmed by ICE and TEE. Despite this finding, arterial pressure, heart rate, and oxygen saturation remained stable, and there were no signs of tamponade or hemodynamic compromise.

At necropsy, the LAA was identified and exhibited a discolored purple ring, most likely reflecting localized bruising and hemorrhage from suction and catheter contact. A small perforation was present in the appendage wall, corresponding to the region of maximal catheter interaction. No large pericardial clot or hemothorax was observed.

## 4. Discussion

This exploratory experiment was designed to assess technical achievability rather than therapeutic efficacy. In this first-in-animal feasibility study, we demonstrated that vacuum-assisted inversion of the LAA can be achieved using a fully catheter-based, transseptal approach. The technique aims to mechanically exclude the appendage by inverting its apex into the left atrium without the need for clips, sutures, or occluder implants [11,12].

The ability to invert the LAA using negative pressure has important translational implications. Existing LAA occlusion strategies rely on permanent devices that may be associated with device-related thrombus, peri-device leaks, and the need for post-procedural antithrombotic therapy. A successful non-implant approach could potentially mitigate these limitations while preserving the minimally invasive nature of contemporary structural heart procedures.

The present work complements the study by Wang et al. [13], which established the anatomical and biological feasibility of LAA inversion in swine using direct mechanical manipulation. Whereas their study focused on the healing response and long-term remodeling of an inverted appendage, our experiment introduces a purely intravascular method capable of generating inversion using tools and workflows familiar to interventional cardiologists. Together, these studies outline a potential pathway from concept to catheter-based therapy. Unlike the chronic inversion model described by Wang et al., the present study was limited to acute intraprocedural assessment and therefore cannot determine whether inversion geometry would remain stable after recovery. Partial or complete reversion of the appendage to its native anatomy remains possible and requires dedicated chronic survival investigation.

**Figure 5:**
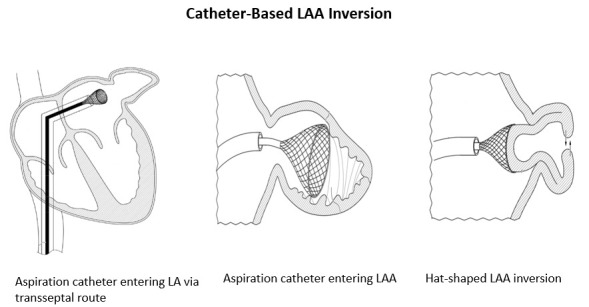
Conceptual schematic illustrating the proposed mechanism of catheter-based vacuum-assisted inversion of the left atrial appendage. After femoral venous access, a delivery catheter is advanced through the inferior vena cava, right atrium, and across the interatrial septum into the left atrium. A funnel-shaped aspiration catheter is positioned at the LAA orifice and directed toward the appendage cavity.

The central illustration (Figure 5) summarizes the mechanistic steps of catheter engagement, suction-induced folding, and complete inversion. The concept aligns with prior surgical inversion studies but introduces a fully percutaneous approach. The observed focal tissue injury and appendage perforation may theoretically create thrombogenic surfaces during healing. Accordingly, future chronic studies must assess endothelialization, thrombus formation, inflammatory response, and embolic risk after inversion.

Several limitations emerged. The aspiration catheter used in this feasibility experiment was not designed specifically for LAA inversion. Its stiff distal tip likely contributed to the observed tissue injury, including local bruising and the small perforation identified at necropsy. Manual generation of negative pressure with a large syringe also limited control over the magnitude and rate of suction, which may have increased mechanical stress on the thin atrial wall.

Future device iterations should incorporate softer atraumatic suction interfaces, pressure-limited suction control, and catheter geometries specifically tailored to conform to left atrial appendage anatomy in order to minimize tissue injury and improve procedural safety. Chronic survival studies in large animals will be essential to determine the durability of inversion, the extent of fibrosis and endothelialization, the impact on atrial function, and the potential for thrombus formation at the inverted stump. Computational modeling of suction forces and wall stress may further inform safe operating parameters.

Despite these limitations, the current study provides an important proof of concept: catheter-based vacuum-assisted LAA inversion is technically achievable. For selected patients with atrial fibrillation who are unsuitable for device-based occlusion or long-term anticoagulation [14,15,16], a refined non-implant inversion strategy may represent a future non-implant therapeutic concept.

## 5. Limitations

This study has several important limitations. First, the findings are derived from a single acute animal experiment and therefore do not establish procedural reproducibility, long-term durability, or safety. Second, the aspiration catheter used in this feasibility study was not specifically designed for left atrial appendage inversion and likely contributed to localized tissue injury. Third, suction was generated manually without pressure-controlled regulation, limiting precise control of applied negative pressure. Finally, chronic follow-up was not performed, and therefore healing response, fibrosis, endothelialization, and thrombotic risk after inversion remain unknown. In addition, the present study cannot determine whether the inverted appendage would maintain stable exclusion over time or gradually revert toward its native configuration.

## 6. Conclusions

This acute first-in-animal experiment demonstrates the technical feasibility of vacuum-assisted catheter-based left atrial appendage inversion using a transseptal catheter approach. The findings should be interpreted as preliminary proof-of-concept observations only. Dedicated atraumatic aspiration devices and chronic survival studies are necessary before therapeutic applicability, long-term durability, or stroke prevention efficacy can be assessed.

## Data Availability

The raw data supporting the conclusions of this article will be made available by the authors on request.
